# Limitations of Nerve Fiber Density as a Prognostic Marker in Predicting Oncological Outcomes in Hepatocellular Carcinoma

**DOI:** 10.3390/cancers14092237

**Published:** 2022-04-29

**Authors:** Jan Bednarsch, Xiuxiang Tan, Zoltan Czigany, Georg Wiltberger, Roman David Buelow, Peter Boor, Sven Arke Lang, Tom Florian Ulmer, Ulf Peter Neumann, Lara Rosaline Heij

**Affiliations:** 1 Department of Surgery and Transplantation, University Hospital RWTH Aachen, 52074 Aachen, Germany; jbednarsch@ukaachen.de (J.B.); xtan@ukaachen.de (X.T.); zczigany@ukaachen.de (Z.C.); gwiltberger@ukaachen.de (G.W.); svlang@ukaachen.de (S.A.L.); fulmer@ukaachen.de (T.F.U.); uneumann@ukaachen.de (U.P.N.); 2 NUTRIM School of Nutrition and Translational Research in Metabolism, Maastricht University, 6229 ER Maastricht, The Netherlands; 3 Institute of Pathology, University Hospital RWTH Aachen, 52074 Aachen, Germany; rbuelow@ukaachen.de (R.D.B.); pboor@ukaachen.de (P.B.); 4 Department of Nephrology and Immunology, University Hospital RWTH Aachen, 52074 Aachen, Germany; 5 Department of Surgery, UMC+ Maastricht University Hospital, 6229 HX Maastricht, The Netherlands

**Keywords:** hepatocellular carcinoma, nerve fibers, tumor microenvironment, oncological outcome, biomarker

## Abstract

**Simple Summary:**

Nerve fibers in the microenvironment have shown notable prognostic potential in various malignancies; however, its role in hepatocellular carcinoma remains to be elucidated. Therefore, the impact of nerve fibers on oncological survival was investigated in a large European cohort of patients with hepatocellular carcinoma who underwent curative-intent liver resection. By means of univariate and multivariate statistics as well as group comparisons of patients with and without nerve fibers, the presence of nerve fibers itself, as well as the corresponding density, was not shown to be associated with survival or the risk of tumor recurrence. Despite being of major prognostic value in various cancer types, nerve fibers in the microenvironment of hepatocellular carcinoma could not be used as a prognostic biomarker in these patients.

**Abstract:**

It has been shown that the presence and density of nerve fibers (NFs; NFD) in the tumor microenvironment (TME) may play an important prognostic role in predicting long-term oncological outcomes in various malignancies. However, the role of NFD in the prognosis of hepatocellular carcinoma (HCC) is yet to be explored. To this end, we aimed to investigate the impact of NFs on oncological outcomes in a large European single-center cohort of HCC patients. In total, 153 HCC patients who underwent partial hepatectomy in a curative-intent setting between 2010 and 2021 at our university hospital were included in this study. Group comparisons between patients with and without NFs were conducted and the association of recurrence-free survival (RFS) and overall survival (OS) with the presence of NFs and other clinico-pathological variables were determined by univariate and multivariable Cox regression models. Patients with NFs in the TME presented with a median OS of 66 months (95% CI: 30–102) compared to 42 months (95% CI: 20–63) for patients without NFs (*p* = 0.804 log-rank). Further, RFS was 26 months (95% CI: 12–40) for patients with NFs compared to 18 months (95% CI: 9–27) for patients without NFs (*p* = 0.666 log-rank). In a subgroup analysis, patients with NFD ≤ 5 showed a median OS of 54 months (95% CI: 11–97) compared to 48 months (95% CI: 0–106) for the group of patients with NFD > 5 (*p* = 0.787 log-rank). Correspondingly, the RFS was 26 months (95% CI: 10–42) in patients with NFD ≤ 5 and 29 months (95% CI: 14–44) for the subcohort with NFD > 5 (*p =* 0.421 log-rank). Further, group comparisons showed no clinico-pathological differences between patients with NFs (*n* = 76) and without NFs (*n* = 77) and NFs were not associated with OS (*p* = 0.806) and RFS (*p* = 0.322) in our Cox regression models. In contrast to observations in various malignancies, NFs in the TME and NFD are not associated with long-term oncological outcomes in HCC patients undergoing surgery.

## 1. Introduction

Hepatocellular carcinoma (HCC) is unquestionably a significant health burden as the third most frequent cause of cancer-related mortality globally [1,2]. Liver resection (LR) remains the primary treatment for patients with early HCC and with an increasingly progressive surgical approach nowadays, resection is often considered in selected patients with advanced tumor stages [3,4,5]. Especially in individuals with limited disease and preserved function of the liver remnant, 10-year survival rates above 50% have been reported in selected cohorts [6]. However, as HCC arises on a background of chronic liver disease, tumor recurrence in the remnant liver is reported in up to 80% of patients, even after complete initial tumor clearance and R0 resection [7]. Based on this, liver transplantation might be the best option for HCC patients as it addresses both the underlying parenchymal liver disease leading to cancer and the oncological disease itself; however, its utilization in this setting is strongly limited by the scarcity of liver allografts from deceased donors [8].

Prognostic biomarkers are important for the development of prognostic models which are suitable to predict clinical prognosis in oncological patients and guide treatment decisions in complex clinical situations [9]. In Europe, HCC treatment is most often guided by the Barcelona Clinic Liver Cancer (BCLC) staging system, which summarizes key prognostic characteristics, such as tumor burden, the extent of liver dysfunction, and the general performance status of the patient [10,11]. However, the overall validity of the rather conservative BCLC staging system has been critically discussed, as some reports indicate a significant survival benefit after liver resection over other interventional or palliative treatment modalities even in higher preoperative BCLC stages [4,5]. Thus, in surgical candidates with HCC, identifying novel prognostic biomarkers and the development of accurate prognostic models are of utmost scientific importance with practice-changing potential.

**Figure 1:**
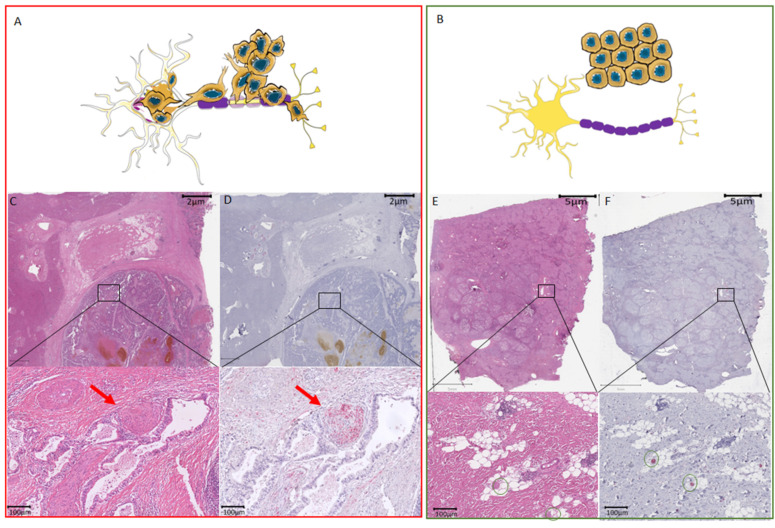
Difference between perineural invasion and nerve fiber density with respect to hepatocellular carcinoma. (**A**) Schematic overview of tissue with cancers cells invading the nerve. (**B**) Schematic overview of tissue with NFs in the TME. (**C**) Routine HE staining showing of HCC. The black box illustrates the zoomed-in area shown underneath with perineural invasion by cancer cells invading a large nerve trunk (red arrow). (**D**) Consecutive slide used for immunohistochemistry with the neuronal marker PGP9.5. The black box illustrates the zoomed-in area shown underneath with perineural invasion by cancer cells invading a large nerve trunk (red arrow). The nerve trunk is illustrated in red. (**E**) Routine HE staining showing of HCC. Routine HE staining with the black box indicates the localization of the small nerve fibers that are not visible on the HE staining. Green circles in the zoomed-in image mark the regions where the small nerve fibers are found by immunohistochemistry. (**F**) Consecutive slide used for immunohistochemistry with the neuronal marker PGP9.5. The black box illustrates the zoomed-in area shown underneath with the presence of small nerve fibers without cancer invasion. These small nerve fibers are illustrated in red and marked by green circles. HCC, hepatocellular carcinoma; NF, nerve fibers; TME, tumor microenvironment.

Our group has recently shown the notable prognostic value of nerve fibers (NFs) in the tumor microenvironment (TME) in cholangiocarcinoma (CCA), which is the second most common primary liver cancer [12,13]. As these NFs have a small diameter (diameters of <100 μm) and are usually not visible on routine H&E staining and require additional immunohistochemical staining (Figure 1). NFs and their respective count (nerve fiber density, NFD) have shown to have a prognostic significance in other malignancies as well (e.g., pancreatic ductal adenocarcinoma (PDAC) and gastric or colorectal cancer) [14,15,16,17], however, their exact role in HCC patients has not been investigated before. Therefore, in this study, we subsequently investigated NFs as a prognostic marker in a large European cohort of HCC patients undergoing hepatectomy with a curative intent.

## 2. Materials and Methods

### 2.1. Patients

All consecutive patients who underwent surgical resection for HCC between 2010 and 2020 at the University Hospital RWTH Aachen (UH-RWTH) were considered for inclusion in this study. Of these patients (*n* = 212), 59 individuals were ultimately excluded (this includes: *n* = 49 with missing NF data; *n* = 10 cases of perioperative mortality), resulting in a study cohort of 153 patients. The study was approved by the institutional Ethical Review Board (EK 106/18) and was carried in line with the good clinical practice guidelines (ICH-GCP) and the principles of the Declaration of Helsinki.

### 2.2. Staging and Surgical Technique

All individuals treated for HCC in our institution underwent a detailed clinical workup as previously described [2,18]. Magnetic resonance imaging (MRI) or computed tomography (CT) was utilized to assess the number, size, and location of tumor nodules as well as the presence of distant metastases. The preoperative risk assessment was based on the American society of anesthesiologists—(ASA) performance status, preoperative calculation of the future liver remnant (FLR) as well as an evaluation of the parenchymal liver function (standard laboratory parameters and the LiMAx test (Humedics^®^ GmbH, Berlin, Germany)) [19]. Patients staged Barcelona Clinic Liver Cancer (BCLC) A to C without any evidence of extrahepatic spread and compensated liver function were considered candidates for surgery as primary treatment. The final decision for surgery was made by a dedicated hepatobiliary surgeon and approved by the local interdisciplinary tumor board for every HCC patient. Liver resection was carried out in line with our clinical standards [2,18]. Briefly, an intraoperative ultrasound was performed regularly to detect other suspicious lesions and visualize the local tumor spread. The decision for either non-anatomic atypical wedge resections with an adequate resection margin or anatomic resections or was based on the surgeon’s preference. Parenchymal transection was carried out with the Cavitron Ultrasonic Surgical Aspirator (CUSA^®^, Integra LifeSciences^®^, Plainsboro, NJ, USA) in open hepatectomy, while in laparoscopic resection, parenchymal transection was commonly performed by Harmonic Ace^®^ (Ethicon Inc., Somerville, NJ, USA), Thunderbeat ^®^ (Olympus K.K., Tokyo, Japan) or laparoscopic CUSA (Integra Life Sciences, Princeton, NJ, USA) in combination with polymer clips (Teleflex Inc., Wayne, PA, USA) or vascular staplers (Echelon, Ethicon, Somerville, NJ, USA). Intermittent Pringle maneuvers were used if necessary. The anesthesiologic management was based on a restrictive fluid intervention strategy ensuring a low central venous pressure (CVP) during parenchymal dissection.

### 2.3. Assessment of Nerve Fibers

The formalin-fixed paraffin-embedded (FFPE) blocks were retrieved from the archive of the local Institute of Pathology. Slides (2.5 μm sections) were cut to conduct immunohistochemistry staining with the neuronal marker PGP9.5. Before this, the tissue was deparaffinized in xylene and rehydrated in graded alcohols. Subsequently, the tissue was heated in citrate buffer (pH 6.0) at 95–100 °C for 5 min and cooled down for 20 min. The immunostaining anti-rabbit PGP9.5 (Dako antibody 1:100) was incubated overnight at 4 °C. A Ventana digital scanner was used to digitalize all slides. The digital image was processed in Qupath 0.1.6. The nerve fiber count was analyzed by a trained pathologist who was blinded to the clinical outcomes in every case. The presence of nerve fascicles with diameters of <100 μm in 20 continuous non-overlapping visual fields at ×200 magnification was subsequently assessed [12,16].

### 2.4. Statistical Analysis

The primary endpoint of this analysis was recurrence-free survival (RFS), which was defined as the period from surgery to the date of the first recurrence. The secondary endpoint was overall survival (OS), which was defined as the time period between the date of resection and the date of death of any cause. Patients not displaying tumor recurrence were censored at the time of death or the last follow-up. Perioperative mortality was defined as in-hospital mortality. Group comparisons were conducted by the chi-squared test, Fisher’s exact test, or linear-by-linear association for categorical variables and by the Mann–Whitney-U-Test in case of continuous variables. The associations of the endpoints with clinico-pathological variables were assessed by a univariate and multivariable Cox regression analyses in a forward selection model. Survival curves were generated according to the Kaplan–Meier method and compared with the log-rank test. Median follow-up was calculated according to the reverse Kaplan–Meier method. The level of significance was *p* < 0.05 and *p*-values were considered for two-sided testing. Analyses were performed using SPSS Statistics 24 (IBM Corp., Armonk, NY, USA).

## 3. Results

### 3.1. Patient Cohort

The study group consisted of 105 men (68.6%) and 48 women (31.4%) with a median age of 69 years. Most individuals were assessed as ASA (American Society of Anesthesiologists classification) III or higher (66.0%, 101/153). Non-alcoholic fatty liver disease (NAFLD, 60/153, 39.2%) was the most common disease etiology, followed by viral hepatitis (39/153, 25.5), alcoholic liver disease (ALD, 34/153, 22.2%), and cryptogenic or other diseases (20/153; 20). Liver function was mainly compensated, with most individuals being diagnosed as Child-Pugh A patients (139/153, 90.85). Most of the patients underwent minor liver resection (95/153, 62.1%). Accordingly, R0 resection was achieved in 96.1% (147/153) of the overall cohort. NFs in the TME were present in 76 individuals (49.7%). Major complications as defined by Clavien-Dindo ≥ IIIa were observed in 21.6% (33/153) of the patients. Patients decreasing due to postoperative complications were excluded from the analysis as stated above. Further details, including preoperative imaging characteristics and clinico-pathological details, are displayed in Table 1.

### 3.2. Survival Analysis with Respect to the Presence of Nerve Fibers in the Tumor Microenvironment and Nerve Fiber Density

**Figure 2:**
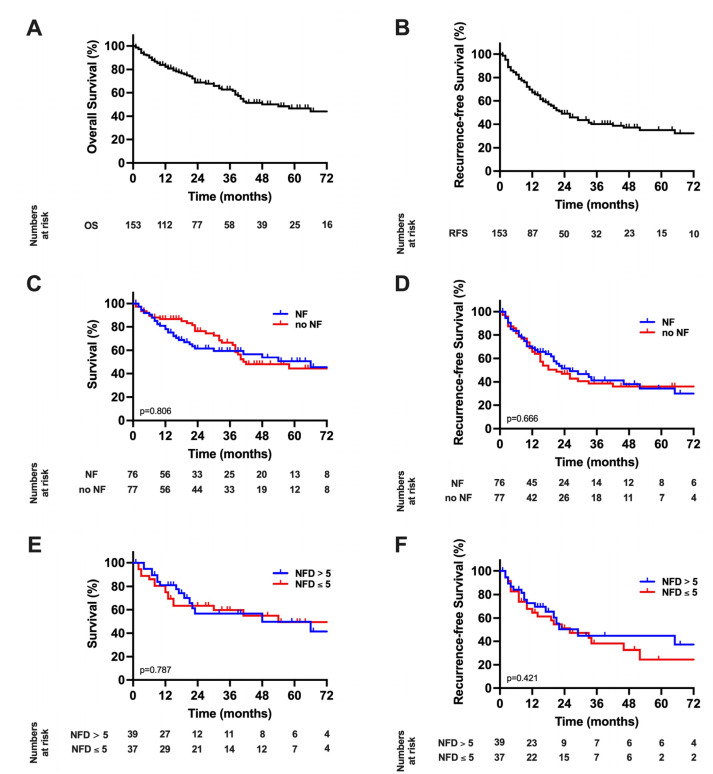
Long-term outcome in hepatocellular carcinoma; (**A**) Overall survival. The median OS of the study cohort was 54 months; (**B**) Recurrence-free survival. The median RFS was 23 months; (**C**) Overall survival stratified by nerve fibers. The median OS of 66 months in patients with NF compared to 42 months (*p* = 0.804 log-rank). (**D**) Recurrence-free survival stratified by nerve fibers. The median RFS was 26 months in patients with NF compared to 18 months in patients without NF (*p* = 0.666 log-rank). (**E**) Overall survival in patients with nerve fibers survival stratified by nerve fiber density. The median OS was 54 months in patients with NFD ≤ 5 compared to 48 months in patients with NFD > 5 (*p* = 0.787 log-rank). (**F**) Recurrence-free survival in patients with nerve fibers survival stratified by nerve fiber density. The RFS was 26 months in patients with NFD ≤ 5 and 29 months patients with NFD > 5 (*p* = 0.421 log-rank). RFS, recurrence-free survival; OS, overall survival.

The median follow-up was calculated to be 48 months for the analysis. The median OS of the overall cohort was 54 months (95% confidence interval (CI): 33–74) and the median RFS 23 months (95% CI: 16–30, Figure 2A,B). A Kaplan–Meier analysis with respect to NFs showed a median OS of 66 months (95% CI: 30–102) in patients with NFs compared to 42 months (95% CI: 20–63) in patients without NFs (*p* = 0.804 log-rank, Figure 2C). Further, RFS was 26 months (95% CI: 12–40) in patients with NFs compared to 18 months (95% CI: 9–27) patients without NFs (*p* = 0.666 log-rank, Figure 2D). Further, a quantitative analysis of patients with NFs was carried out by dividing this subgroup into patients demonstrating an NFD of ≤5 compared to >5. Here the median OS was 54 months (95% CI: 11–97) in patients with NFD ≤ 5 compared to 48 months (95% CI: 0–106) in patients with NFD > 5 (*p* = 0.787 log-rank, Figure 2E). Correspondingly, the RFS was 26 months (95% CI: 10–42) in patients with NFD ≤ 5 and 29 months (95% CI: 14–44) patients with NFD > 5 (*p* = 0.421 log-rank, Figure 2F).

To further investigate patients displaying tumor recurrence, we separately analyzed patients with early recurrence (RFS < 24 months, *n* = 66) and late recurrence (RFS ≥ 24 months, *n* = 14). Here no difference in the likelihood of having NFs in the TME was shown between patients with early (31/66, 47.0%) or late (9/14, 64.3%) recurrence (*p* = 0.239). Moreover, separate survival analyses in patients with early and late recurrence showed no influence of the presence of NFs on OS (*p* = 0.182 log-rank; *p* = 0.867 log-rank) or RFS (*p* = 0.800 log-rank; *p* = 0.697 log-rank) in either of the subgroups (Supplementary Figure S1).

In another sub-analysis, we investigated the role of NFs in different disease etiologies. Here no difference in the likelihood of having NFs in the TME was shown between patients with alcoholic (15/34, 44.1%), non-alcoholic fatty (28/60, 46.7%), viral (23/39, 59.0%), and cryptogenic/other liver disease (10/20, 50.0%, *p* = 0.575). Moreover, separate survival analyses for each disease entity showed no influence of the presence of NFs on OS or RFS in either of the underlying liver diseases (Supplementary Figure S2).

### 3.3. Cox Regression Analysis of the Overall Cohort

As neither the presence of NFs in the whole cohort nor NFD within patients displaying NFs in the TME were found to be of prognostic value for OS and RFS in the Kaplan-Meier analysis, Cox regressions were used to determine risk factors for inferior oncological outcomes.

In univariate analysis, gender (*p =* 0.035), ASA score (*p =* 0.011), MELD (*p =* 0.017) AFP, *p =* 0.002) as well as a variety of other liver function parameters and preoperative imaging features, R1 resection (*p =* 0.003), pT category (*p* < 0.001), microvascular invasion (MVI, *p* < 0.001) and the duration of hospitalization (*p =* 0.003) gained statistical significance for OS (Table 2). These variables were transferred to a multivariable Cox regression model. Here, MELD score (*p =* 0.032), number of nodules (*p =* 0.010), preoperative ascites (*p =* 0.022), R1 resection (*p =* 0.002) and MVI (*p* < 0.001) were identified as independent predictors of OS (Table 2). The presence of NFs was not associated with OS in this analysis (*p =* 0.806). A similar approach was conducted for RFS. As for OS a variety of liver function parameters and variables regarding preoperative imaging as well as R1 resection (*p* < 0.001), pT category (*p* < 0.001) and MVI (*p* < 0.001) were significantly associated with RFS (Table 3). These variables were subsequently transferred to a multivariable Cox regression model. Here, aspartate aminotransferase (AST, *p =* 0.005), portal vein invasion (*p =* 0.030), pT category (*p* < 0.001 were independently prognostic for RFS (Table 3). Again, the presence of NFs was not associated with RFS in this analysis (*p =* 0.322).

### 3.4. Comparative Analysis of the Overall Patient Cohort with Respect to Nerve Fibers

To ensure that the presence of NFs was not unequally distributed among other oncological risk factors, a comparative analysis of patients with and without NFs was conducted (Table 1). This comparative analysis revealed no statistical significance in any characteristic and especially no difference was observed in the oncological risk factors of the overall cohort as determined by the univariate or multivariate Cox regression analysis.

### 3.5. Histological Characteristics

H&E and PGP9.5 scans were descriptively analyzed. The tumor region on the H&E staining was also identified on the PGP immunostaining (Figure 1). Nerve fibers in the TME were detected and counted as described previously [12,13].

## 4. Discussion

Chronic liver disease accounts for over 2 million deaths yearly, while HCC is one of the major oncological burdens from a global perspective and is projected to be responsible for more than 1 million annual deaths by 2030 [22]. With a poor overall 5-year survival of less than 20%, it belongs to the most lethal oncological diseases [9]. In this context, biomarkers with strong prognostic value and validity are under the spotlight of clinical and scientific interest as they might help to guide clinical decisions. Therefore, here we analyzed the prognostic value of the novel biomarker NFD within a large European single-center cohort of HCC patients undergoing curative-intent surgery. However, our data did not show any prognostic value for the presence of NFs in the TME or NFD in quantitative or qualitative analysis.

NFs play an integral role in the intense crosstalk of cancer-associated fibroblasts (CAF) or immune cells, or with tumor cells [23,24,25,26]. This inter-cellular crosstalk is partly based on released neurotransmitters of cancer cells binding to receptors of NFs and vice versa [27,28,29,30]. CAF triggers remodeling of the extracellular matrix, resulting in further neuronal growth, ultimately enhancing these effects [23,24]. It should be noted that these particular NFs are speculated to have a parasympathetic origin and must be differentiated from larger preexisting nerve trunks used to define classical PNI [12,12,31,32]. The underlying role of the nervous system in tumorigenesis and disease progression remains to be unraveled. However, some basic research findings did suggest certain antitumoral effects exerted by the parasympathetic system leading to decreased local tumor progression and attenuation of the development of distant metastases [33,34]. Considering these effects, NFs and NFD have been investigated in various oncological diseases and clinical settings, and so far, their prognostic value in predicting oncological outcomes has been reported in gastric and colorectal adenocarcinomas, breast cancer, PDAC as well as in intrahepatic and perihilar CCA [12,12,13,14,15,16,17,35]. Interestingly, seemingly there are disease-related differences regarding the exact role NFs are playing in outcomes. While a high NFD was shown to be associated with an impaired outcome in gastric and colorectal adenocarcinomas, it seems to be protective in terms of long-term survival in PDAC and both subtypes of CCA [12,12,13,14,15,16,17,35]. There are further heterogeneities even in entities with a positive correlation between NFD and oncological endpoints and cut-off values ideally used for differentiation between low-risk and high-risk patients vary between different tumors. While the mere presence or absence of NFs in intrahepatic CCA (NFD > 0) was defined as the best cut-off for defining prognosis, NFD ≥ 10 was determined for perihilar CCA and NFD > 7 for PDAC [12,13,16].

Given the significant prognostic value of NFD in CCA as the second most common liver tumor, we hypothesized that NF or NFD might also be associated with outcomes in HCC. However, our detailed analysis in this study could not find any difference in OS for HCC patients. The underlying mechanistic explanation for this observation is beyond the scope of this study and should be addressed in the future. One might argue that OS in HCC is also heavily influenced by the progression of the underlying liver disease and cirrhosis-associated complications, e.g., hepatic decompensations, malnutrition, sarcopenia, bacterial infections, or variceal bleedings [2,36]. This is an important cofounder which might influence our results as NFs are obviously associated with oncological prognosis in other tumors but not with the severity of cirrhosis. Thus, we decided to define RFS as the primary endpoint of the study and found no association between RFS and NFs, supporting the assumption that NFs might not be of prognostic value in HCC. As stated above, the definition of ideal cut-offs is a topic of ongoing debate and might be different among oncological entities [12,13,16]. Therefore, we conducted a secondary analysis exclusively for patients with NFs in the TME and stratified this subcohort based on the median NFD of these individuals. As we could not detect any tendency for improved OS or RFS in patients with high NFD, we waived a receiver operating characteristic (ROC)-based approach for optimal cut-off detection, which we previously utilized for CCA [12,13]. To also ensure that oncological risk factors of the analyzed study cohort were not unequally distributed between patients with and without NFs, we conducted Cox regression analyses to identify prognostic variables of the cohort and compared patients with NFs to patients without NFs within a group comparison. Here, no between-group differences were detected and no association in the Cox regressions were observed.

As stated above, tumor recurrence might be more suited to identify oncological risk factors as a large set of patients decease due to progression or complications of the underlying liver disease [2,36]. Recurrence patterns of HCC vary among patients, with individuals displaying an early recurrence (usually due to initial multicentric carcinogenesis or early metastatic recurrence) and others suffering from late tumor recurrence (de-novo HCC due to the carcinogenic potential of the underlying liver disease). To back up our observations, we conducted survival analyses for each of these recurrence sub-groups, and neither found a difference in the likelihood of having NFs in the TME between the sub-groups nor found an influence of the presence of NFs on OS or RFS in either of the sub-groups. Thus, we believe that different recurrence patterns do not affect our overall finding that NFs in the TME are not associated with oncological endpoints in HCC.

While our multivariable model identified MELD score, multifocal disease, preoperative ascites, R1 resections, and MVI as independent prognostic characteristics for OS; AST, portal vein invasion, and pT category were identified as independent predictors of RFS. MVI invasion as well as MELD score are known risk factors in HCC and are associated with poor OS [37,38]. Nodule count and vascular invasion are also the mainstays of the Milan criteria, which are used for therapy selection in these patients [39,40]. All parameters shown to be of significant prognostic value are well-known for their respective role in HCC. This underlines the validity of our cohort [10]. While our cohort certainly covers a representative spectrum of surgical candidates with HCC, including patients staged BCLC 0 to C, multifocal disease in one-third of all individuals, different stages of liver dysfunction, and various underlying liver diseases, e.g., viral, ALD and NAFLD, one might argue that our sample size might not be large enough to detect the effects of NFs in HCC. In the previous literature, cohorts of similar sizes were used to assess the prognostic potentials of NFs. Zhao et al. investigated NFs in breast cancer in 144 patients, Iwasaki et al. researched NFD in 256 PDAC patients and our group reported the role of NF in 101 patients with extrahepatic CCA and 95 intrahepatic CCA [12,13,16,35]. Given our sample size of 153 HCC cases and our detailed analysis, we, therefore, consider NFs in the TME and NFD as the corresponding number count not to be associated with OS and RFS in our cohort of HCC patients undergoing liver resection.

The underlying reason for this negative observation remains to be investigated. CCA and PDAC are reported to be neurotropic cancers with a high rate of PNI [41]. PNI can also be observed in colorectal and breast cancer [42,43], while HCC is not a disease to commonly show this histological feature. In neurotropic cancers, nerves are considered a potential pathway for cancer cell dissemination and metastasis in the same way as it is known for the vascular and lymphatic channels [44]. However, as stated above, the nerve fibers considered in determining NFD are independent of the large nerve trunks used to define PNI and require additional staining (PGP9.5) to be revealed. It is speculative whether and how these “protective” NFs interact with the larger nerve trunks, which are prone to tumor infiltration. Moreover, for example, PDAC and CCA are characterized by a prominent desmoplastic and hypovascularized stroma contributing to the unique TME of these tumor entities, which is not seen in HCCs [45]. NF directly regulates stromal compartments in the TME [46]. Here NFs promote angiogenesis and interact with immune cells in the TME on multiple levels [46]. Therefore, it seems plausible that the “protective” effect of NFs requires tumors with large stroma parts, e.g., CCA, to interact with stromal compartments to manifest oncological effects. However, exploring the role of the nervous system in tumorigenesis, cancer progression, and long-term outcome is still in its infancy [46]. The same accounts for our descriptive data and clinical observation, which warrant further translational and preclinical research to explore the underlying mechanism behind the oncological effects of NFs in the TME to explain why our study has failed to show an association between NFs and outcome in HCC.

As with all observational clinical studies‚ our analysis has some inherent limitations. All patients treated for HCC in this study underwent surgery and the corresponding treatment in a monocentric setting, reflecting the authors’ distinct approach to this malignancy. Further, our data is retrospective in nature as this study was not carried out in a controlled clinical trial setting. Moreover, histological data were not available for every consecutive patient of our hepatobiliary center, limiting the final dataset. This limited data set did not allow one to conduct a sub-analysis for histological subtypes. However, our analysis included a distinct analysis with respect to disease etiology, which might indicate some known histological subtypes, e.g., steatohepatitic HCC in NAFLD/non-alcoholic steatohepatitis (NASH) or macrotrabecular HCC in viral hepatitis. Future research regarding NFs in the TME in HCC should further explore the role in various histological subtypes and should also comprise molecular characteristics. As this kind of analysis requires a significant dataset, it is likely that a multicentric approach will be necessary. Another limitation might be that only one pathologist scored the tissue slides for NFs in the TME.

## 5. Conclusions

Notwithstanding the above-mentioned limitations, we have demonstrated for the first time that NFs in the TME of HCC are not associated with long-term outcomes in HCC. These observations underline a major difference compared to studies on other gastrointestinal cancers and warrant further basic research. Large-scale multi-center studies are required to validate and confirm our findings.

## Figures and Tables

**Table 1:** Patients’ characteristics.

Variables	Overall Cohort (*n* = 153)	NF Positive (*n* = 76)	NF Negative (*n* = 77)	*p* Value
Demographics				
Gender, m/f (%)	105 (68.6)/48 (31.4)	47 (61.8)/29 (38.2)	58 (75.3)/19 (24.7)	0.072
Age (years)	69 (61–75)	68 (59–75)	70 (63–76)	0.071
BMI (kg/m^2^)	26 (23–29)	26 (23–29)	27 (23–30)	0.512
Recurrence resection, *n* (%)	13 (8.5)	9 (11.8)	4 (5.2)	0.374
Preoperative treatment				
Preoperative PVE, *n* (%)	7 (4.6)	3 (3.9)	4 (5.2)	0.712
Preoperative TACE, *n* (%)	8 (5.2)	4 (5.3)	4 (5.2)	0.985
Preoperative TARE, *n* (%)	2 (1.3)	2 (2.6)	0	0.152
ASA, *n* (%)				0.508
I	2 (1.3)	2 (2.6)	0	
II	50 (32.7)	23 (30.3)	27 (35.1)	
III	97 (63.4)	49 (64.5)	48 (62.3)	
IV	4 (2.6)	2 (2.6)	2 (2.6)	
V	0	0	0	
Liver disease, *n* (%)				0.575
ALD	34 (22.2)	15 (19.7)	19 (24.7)	
NAFLD	60 (39.2)	28 (36.8)	32 (41.6)	
Viral	39 (25.5)	23 (30.3)	16 (20.8)	
Cryptogenic/others	20 (13.1)	10 (13.2)	10 (13.0)	
Preoperative liver function				
MELD Score	6 (6–7)	6 (6–7)	6 (6–7)	0.965
AFP (ng/mL)	8 (3–53)	11 (3–95)	6 (3–45)	0.202
Albumin (g/dL)	4.1 (3.7–4.5)	4.1 (3.6–4.4)	4.1 (3.8–4.5)	0.269
AST (U/L)	40 (27–58)	40 (28–63)	38 (26–58)	0.374
ALT (U/L)	33 (23–54)	37 (25–58)	30 (21–51)	0.123
GGT (U/L)	90 (51–213)	92 (55–178)	90 (50–267)	0.822
Total bilirubin (mg/dL)	0.5 (0.4–0.8)	0.5 (0.4–0.8)	0.6 (0.4–0.8)	0.515
Platelet count (/nL)	221 (163–279)	225 (161–278)	206 (168–282)	0.818
Alkaline Phosphatase (U/L)	100 (77–140)	95 (75–180)	103 (79–134)	0.860
Prothrombin time (%)	93 (85–101)	92 (83–104)	93 (85–100)	0.868
INR	1.05 (0.98–1.11)	1.05 (0.98–1.10)	1.04 (0.98–1.11)	0.898
Creatinine (mg/dL)	0.85 (0.70–1.04)	0.84 (0.70–1.01)	0.87 (0.72–1.09)	0.526
Hemoglobin (g/dL)	13.2 (11.7–14.4)	12.8 (11.7–14.1)	13.5 (11.9–14.8)	0.131
Child-Pugh, *n* (%)				0.088
A	139 (90.8)	66 (86.8)	73 (94.8)	
B	14 (9.2)	10 (13.2)	4 (5.2)	
Preoperative Imaging features				
Number of nodules	1 (1–2)	1 (1–2)	1 (1–2)	0.324
Largest nodule diameter (mm)	50 (32–80)	49 (32–78)	53 (34–84)	0.340
Tumor burden >50%, *n* (%)	7 (4.6)	4 (5.3)	3 (3.9)	0.686
Overall macrovascular invasion, *n* (%)	39 (25.5)	21 (27.6)	18 (23.4)	0.546
Portal vein invasion, *n* (%)	24 (15.7)	14 (18.4)	10 (13.0)	0.355
Extrahepatic vascular invasion, *n* (%)	8 (5.2)	3 (3.9)	5 (6.5)	0.479
Portal vein thrombosis, *n* (%)	6 (3.9)	3 (3.9)	3 (3.9)	0.987
Ascites, *n* (%)	6 (3.9)	3 (3.9)	3 (3.9)	0.987
BCLC, *n* (%)				0.709
0	7 (4.6)	4 (5.3)	3 (3.9)	
A	89 (58.2)	41 (53.9)	48 (62.3)	
B	33 (21.6)	17 (22.4)	16 (20.8)	
C	24 (15.7)	14 (18.4)	10 (13.0)	
D	0	0	0	
Operative Data				
Laparoscopic resection, *n* (%)	58 (37.9)	28 (36.8)	30 (39.0)	0.787
Conversation rate, *n* (%)	5 (8.6)	2 (7.1)	3 (10.0)	0.698
Operative time (minutes)	204 (146–274)	206 (140–274)	199 (150–273)	0.469
Operative procedure, *n* (%)				0.575
Atypical	59 (38.6)	27 (35.5)	32 (41.6)	
Segmentectomy	21 (13.7)	8 (10.5)	13 (16.9)	
Bisegmentectomy	15 (9.8)	8 (10.5)	7 (9.1)	
Hemihepatectomy	34 (22.2)	17 (22.4)	17 (22.1)	
Extended liver resection	17 (11.1)	12 (15.8)	5 (6.5)	
ALPPS/TSH/other	7 (4.6)	4 (5.2)	3 (3.9)	
Additional procedures (RFA, etc.), *n* (%)	7 (4.6)	3 (3.9)	4 (5.2)	0.712
Pringle maneuver, *n* (%)	10 (6.6)	4 (5.3)	6 (7.9)	0.513
Duration of pringle maneuver (min) *	18 (10–24)	11 (6–33)	20 (14–24)	0.352
Intraoperative blood transfusion, *n* (%)	42 (28.0)	21 (27.6)	21 (27.6)	0.919
Intraoperative FFP, *n* (%)	58 (38.7)	25 (33.8)	33 (43.4)	0.226
Intraoperative platelet transfusion, *n* (%)	4 (2.7)	1 (1.4)	3 (3.9)	0.324
Pathological examination				
R0 resection, *n* (%)	147 (96.1)	75 (98.7)	72 (93.5)	0.099
T category, *n* (%)				0.532
T1	67 (34.8)	36 (47.4)	31 (40.3)	
T2	57 (37.3)	25 (32.9)	32 (41.6)	
T3/T4	29 (19.0)	15 (19.7)	14 (18.2)	
Microvascular invasion, *n* (%)	62 (44.0)	32 (45.1)	30 (42.9)	0.791
Tumor grading, *n* (%)				0.253
G1/G2	122 (80.3)	63 (84.0)	59 (76.6)	
G3/G4	30 (19.7)	12 (16.0)	18 (23.4)	
NF, *n* (%)	76 (49.7)	76 (100)	0	<0.001
NFD	0 (0–5)	6 (2–10)	0 (0–0)	<0.001
Postoperative Data				
Intensive care stay, days	1 (1–1)	1 (1–1)	1 (1–1)	0.946
Hospitalization, days	8 (6–8)	8 (5–15)	8 (6–13)	0.772
Postoperative complications, *n* (%)				0.520
No complications	81 (52.9)	38 (50.0)	43 (55.8)	
Clavien-Dindo I	15 (9.8)	11 (14.5)	4 (5.2)	
Clavien-Dindo II	24 (15.7)	12 (15.8)	12 (15.6)	
Clavien-Dindo IIIa	19 (12.4)	8 (10.5)	11 (14.3)	
Clavien-Dindo IIIb	7 (4.6)	4 (5.3)	3 (3.9)	
Clavien-Dindo IVa	6 (3.9)	3 (3.9)	3 (3.9)	
Clavien-Dindo IVb	1 (0.7)	0	1 (1.3)	
Clavien-Dindo V	0	0	0	
PHLF 50-50 criteria *, *n* (%)	0	0	0	n.a.
PHLF ISGLS *, *n* (%)	25 (16.3)	12 (15.8)	13 (16.9)	0.855
ISGLS Grade, *n* (%)				0.755
A	20 (80.0)	9 (75.0)	11 (84.6)	
B	4 (16.0)	2 (16.7)	2 (15.4)	
C	1 (4.0)	1 (8.3)	0	
Postoperative blood transfusion	19 (12.7)	8 (10.8)	11 (14.5)	0.500
Postoperative FFP	6 (4.0)	4 (5.4)	2 (2.6)	0.386
Postoperative platelet transfusion	1 (0.7)	1 (1.4)	1 (1.3)	0.309
Follow-up Data				
Recurrence-free survival (months)	23 (16–30)	26 (12–40)	18 (9–27)	0.666
Overall survival (months)	54 (34–74)	66 (30–102)	42 (21–63)	0.804

Data presented as median and interquartile range if not indicated otherwise. Long-term outcome data are presented as median and 95% CI. Chi-squared test, fisher’s exact test, or linear-by-linear association were used to compare categorical data. The Mann–Whitney-U-Test was used to compare continuous data. * Postoperative liver failure was assessed by the 50-50-criteria and the ISGLS definition [20,21]. ALD, alcoholic liver disease; ALPPS; Associating liver partition with portal vein ligation for staged hepatectomy; ALT, alanine aminotransferase; AP, alkaline phosphatase; ASA, American society of anesthesiologists classification; AST, aspartate aminotransferase; BCLC, Barcelona clinical liver cancer staging system; BMI, body mass index; CI, confidence interval. FFP, fresh frozen plasma; GGT, gamma-glutamyltransferase; INR, international normalized ratio; ISGLS, International Study Group of Liver Surgery; MELD, model of end-stage liver disease; NAFLD, Non-alcoholic fatty liver disease; NF, nerve fibers; NFD, nerve fiber density; PHLF, Posthepatectomy liver failure; PVE; portal vein embolization; RFA, radiofrequency ablation; TACE, transarterial chemoembolization; TARE, transarterial radioembolization; TSH, Two-stage hepatectomy.

**Table 2:** Univariate and multivariable analysis of overall survival in hepatocellular carcinoma.

Variables	Univariate Analysis		Multivariable Analysis	
	HR (95% CI)	*p*-Value	HR (95% CI)	*p*-Value
Demographics				
Gender (male = 1)	1.82 (1.04–3.16)	0.035		0.123
Age (≤65 years = 1)	1.03 (0.62–1.70)	0.915		
BMI (≤25 kg/m^2^ = 1)	0.95 (0.58–1.57)	0.851		
Recurrence resection (no = 1)	0.62 (0.20–1.99)	0.424		
ASA (I/II = 1)	2.09 (1.18–3.69)	0.011		0.188
Liver disease		0.116		
ALD	1			
NAFLD	0.52 (0.28–0.96)			
Viral	0.65 (0.33–1.21)			
Cryptogenic/others	0.41 (0.16–1.04)			
Preoperative liver function				
MELD Score (≤6 = 1)	1.91 (1.12–3.24)	0.017	2.08 (1.07–4.05)	0.032
Albumin (≤40 g/L = 1)	0.57 (0.34–0.94)	0.027		0.726
AFP (≤10 µg/L = 1)	2.56 (1.40–4.67)	0.002		excl.
AST (≤40 U/L = 1)	1.89 (1.12–3.18)	0.016		0.551
ALT (≤40 U/L = 1)	1.64 (0.94–2.84)	0.079		
GGT (≤100 U/L = 1)	2.66 (1.54–4.60)	<0.001		0.354
Bilirubin (≤1 mg/dL = 1)	1.82 (0.96–3.43)	0.066		
AP (≤100 U/L = 1)	1.95 (1.16–3.28)	0.011		0.462
Platelet count (≤200/nL = 1)	1.00 (0.60–1.65)	0.988		
INR (≤1 = 1)	1.82 (1.03–3.20)	0.039		0.130
Creatinine (≤1 = 1)	1.19 (0.70–2.02)	0.531		
Hemoglobin (≤12 g/dL = 1)	0.80 (0.48–1.34)	0.399		
Child Pugh (A = 1)	2.96 (1.44–6.07)	0.003		0.556
Preoperative Imaging features				
Number of nodules (1 = 1)	3.20 (1.95–5.24)	<0.001	2.01 (1.20–4.05)	0.010
Largest nodule diameter (≤50 mm = 1)	1.89 (1.14–3.13)	0.013		0.405
Tumor burden (≤50% = 1)	2.90 (1.25–6.76)	0.014		0.484
Macrovascular invasion (no = 1)	2.23 (1.33–3.71)	0.002		0.999
Portal vein invasion (no = 1)	2.88 (1.63–5.09)	<0.001		0.084
Extrahepatic vascular invasion (no = 1)	2.40 (1.03–5.59)	0.042		0.700
Portal vein thrombosis (no = 1)	3.09 (1.23–7.72)	0.016		0.117
Ascites (no = 1)	3.24 (1.15–9.09)	0.025	6.24 (1.30–29.98)	0.022
BCLC		<0.001		0.190
0/A	1			
B	3.17 (1.80–5.58)			
C	4.30 (2.29–8.07)			
Operative Data				
Laparoscopic resection (no = 1)	1.94 (1.05–3.58)	0.034		0.664
Operative time (≤180 min = 1)	1.44 (0.86–2.41)	0.163		
Operative procedure (minor = 1)	1.15 (0.70–1.87)	0.588		
Additional procedures (no = 1)	1.25 (0.39–4.02)	0.705		
Pringle maneuver (yes = 1)	0.56 (0.24–1.32)	0.185		
Intraop blood transfusion (no = 1)	1.50 (0.89–2.53)	0.128		
Intraop FFP (no = 1)	1.36 (0.83–2.23)	0.219		
Pathological data				
R1 resection (no = 1)	3.58 (1.54–8.33)	0.003	5.52 (1.86–16.38)	0.002
pT category		<0.001		0.192
T1	1			
T2	2.86 (1.51–5.43)			
T3/T4	6.19 (3.15–12.18)			
Tumor grading (G1/G2 = 1)	1.41 (0.79–2.51)	0.248		
MVI (no = 1)	4.27 (2.39–7.63)	<0.001	4.27 (2.18–8.37)	<0.001
NF (no = 1)	1.06 (0.65–1.73)	0.806		
Postoperative Data				
Intensive care stay (≤1 day = 1)	1.19 (0.57–2.51)	0.641		
Hospitalization (≤7 days = 1)	2.44 (1.35–4.42)	0.003		0.094
Postop complications (I/II = 1)	1.22 (0.70–2.13)	0.482		
PHLF ISGLS (no = 1)	1.14 (0.62–2.09)	0.682		
Postop blood transfusion (no = 1)	1.33 (0.67–2.61)	0.414		
Postop FFP (no = 1)	0.51 (0.12–2.09)	0.348		

Various parameters are prognostic for overall survival. AFP, alpha-fetoprotein; ALD, alcoholic liver disease; ALT, alanine aminotransferase; AP, Alkaline phosphatase; ASA, American society of anesthesiologists classification; AST, aspartate aminotransferase; BCLC, Barcelona clinical liver cancer staging system; BMI, body mass index; CI, confidence interval. FFP, fresh frozen plasma; GGT, gamma-glutamyltransferase; INR, international normalized ratio; ISGLS, International Study Group of Liver Surgery; MELD, model of end-stage liver disease; NAFLD, Non-alcoholic fatty liver disease; NF, nerve fibers; PHLF, Posthepatectomy liver failure.

**Table 3:** Univariate and multivariable analysis of recurrence-free survival in hepatocellular carcinoma. Various parameters are prognostic for recurrence-free survival.

	Univariate Analysis		Multivariable Analysis	
	HR (95% CI)	*p*-Value	HR (95% CI)	*p*-Value
Demographics				
Gender (male = 1)	1.00 (0.63–1.58)	0.985		
Age (≤65 years = 1)	0.77 (0.49–1.20)	0.249		
BMI (≤25 kg/m^2^ = 1)	0.83 (0.54–1.30)	0.830		
Recurrence resection (no = 1)	1.07 (0.49–2.33)	0.863		
ASA (I/II = 1)	1.05 (0.67–1.66)	0.836		
Liver disease		0.316		
ALD	1			
NAFLD	0.63 (0.35–1.14)			
Viral	1.00 (0.55–1.79)			
Cryptogenic/others	0.77 (0.35–1.71)			
Preoperative liver function				
MELD Score (≤6 = 1)				
Albumin (≤40 g/L = 1)	1.42 (0.88–2.33)	0.155		
AFP (≤10 µg/L = 1)	0.91 (0.58–1.41)	0.662		
AST (≤40 U/L = 1)	2.15 (1.29–3.57)	0.003		Excl.
ALT (≤40 U/L = 1)	2.45 (1.53–3.93)	<0.001	2.35 (1.30–4.25)	0.005
GGT (≤100 U/L = 1)	2.05 (1.25–3.36)	0.005		0.743
Bilirubin (≤1 mg/dL = 1)	1.84 (1.15–2.93)	0.011		0.303
AP (≤100 U/L = 1)	1.77 (0.97–3.23)	0.062		
Platelet count (≤200/nL = 1)	1.85 (1.17–2.92)	0.009		0.215
INR (≤1 = 1)	0.90 (0.57–1.41)	0.631		
Creatinine (≤1 = 1)	1.50 (0.92–2.45)	0.108		
Hemoglobin (≤12 g/dL = 1)	0.77 (0.46–1.26)	0.297		
Child Pugh (A = 1)	0.79 (0.50–1.26)	0.330		
Preoperative Imaging features	2.20 (1.00–4.84)	0.050		
Number of nodules (1 = 1)				
Largest nodule diameter (≤ 50 mm = 1)				
Tumor burden (≤50% = 1)	3.78 (2.38–6.00)	<0.001		0.663
Macrovascular invasion (no = 1)	1.76 (1.13–2.74)	0.013		0.519
Portal vein invasion (no = 1)	2.39 (0.96–5.96)	0.061		
Extrahepatic vascular invasion (no = 1)	1.93 (1.19–3.13)	0.007		0.669
Portal vein thrombosis (no = 1)	2.42 (1.37–4.26)	0.002	2.44 (1.09–5.45)	0.030
Ascites (no = 1)	2.48 (0.99–6.20)	0.051		
BCLC	5.90 (2.06–16.91)	0.001		0.689
0/A	1.34 (0.33–5.51)	0.685		
B		<0.001		0.725
C	1			
Operative Data	3.13 (1.89–5.19)			
Laparoscopic resection (no = 1)	3.42 (1.86–6.26)			
Operative time (≤180 min = 1)				
Operative procedure (minor = 1)				
Additional procedures (no = 1)	1.32 (0.81–2.16)	0.263		
Pringle maneuver (yes = 1)	1.25 (0.80–1.97)	0.327		
Intraop blood transfusion (no = 1)	1.29 (0.83–2.02)	0.259		
Intraop FFP (no = 1)	1.30 (0.47–3.55)	0.616		
Pathological data	0.55 (0.24–1.26)	0.156		
R1 resection (no = 1)	1.23 (0.75–2.00)	0.414		
pT category	1.07 (0.68–1.68)	0.784		
T1				
T2	4.91 (2.10–11.49)	<0.001		0.243
T3/T4		<0.001		<0.001
Tumor grading (G1/G2 = 1)	1		1	
MVI (no = 1)	3.28 (1.91–5.64)		6.04 (2.89–12.60)	
NF (no = 1)	5.98 (3.15–11.38)		6.02 (2.35–15.43)	
Postoperative Data	1.18 (0.68–2.04)	0.565		
Intensive care stay (≤1 day = 1)	2.38 (1.55–3.96)	<0.001		0.897
Hospitalization (≤7 days = 1)	0.80 (0.52–1.24)	0.322		
Postop complications (I/II = 1)				
PHLF ISGLS (no = 1)	1.22 (0.64–2.31)	0.547		
Postop blood transfusion (no = 1)	1.14 (0.72–1.79)	0.572		
Postop FFP (no = 1)	0.92 (0.52–1.62)	0.774		

AFP, alpha-fetoprotein; ALD, alcoholic liver disease; ALT, alanine aminotransferase; AP, Alkaline phosphatase; ASA, American society of anesthesiologists classification; AST, aspartate aminotransferase; BCLC, Barcelona clinical liver cancer staging system; BMI, body mass index; CI, confidence interval. FFP, fresh frozen plasma; GGT, gamma-glutamyltransferase; INR, international normalized ratio; ISGLS, International Study Group of Liver Surgery; MELD, model of end-stage liver disease; NAFLD, Non-alcoholic fatty liver disease; NF, nerve fibers; PHLF, Posthepatectomy liver failure.

## Data Availability

The data presented in this study are available on request from the corresponding author.

## Supplementary Materials

The following supporting information can be downloaded at: https://www.mdpi.com/article/10.3390/cancers14092237/s1, Figure S1: Outcome in hepatocellular carcinoma with respect to early and late tumor recurrence. Figure S2: Outcome in hepatocellular carcinoma with respect disease etiology.
