# Supplementary material for: Limitations of Nerve Fiber Density as a Prognostic Marker in Predicting Oncological Outcomes in Hepatocellular Carcinoma
Source: Cancers (Basel). 2022 Apr 29;14(9):2237. doi: 10.3390/cancers14092237 (PMC9103173; doi:10.3390/cancers14092237)
Supplement: Supplementary file 1 [file cancers-14-02237-s001.zip › cancers-1647430-supplementary.pdf]

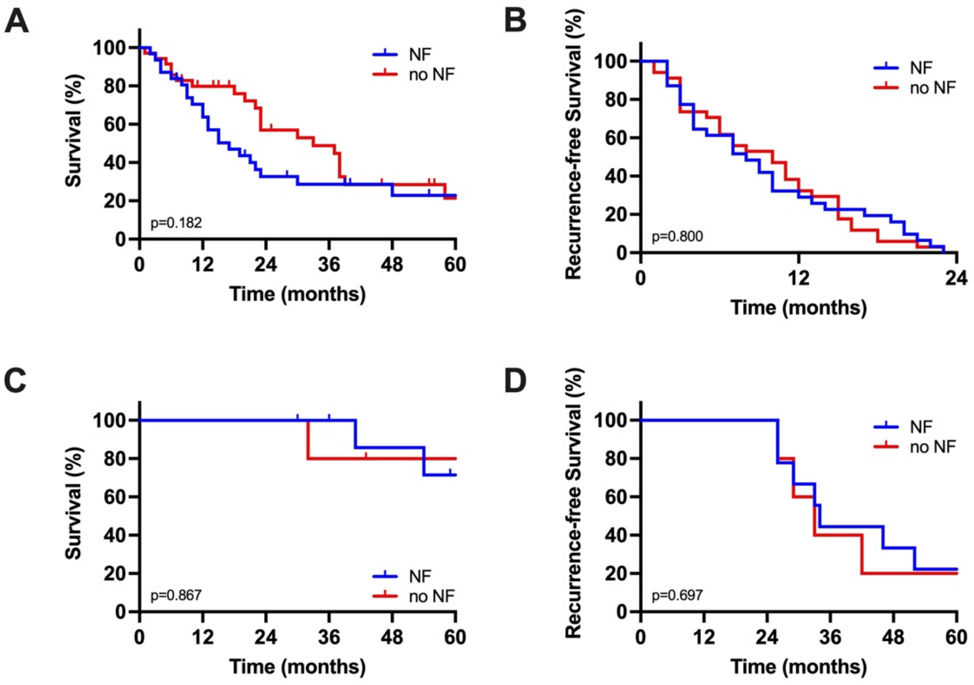

Supplementary Figure S1. Outcome in hepatocellular carcinoma with respect to early and late tumor recurrence. A: Overall survival in patients with early recurrence stratified by nerve fibers. The median OS was 17 months in patients with NF compared to 33 months in patients without NF (p=0.182 log rank). **B: Recurrence-free survival in patients with early recurrence stratified by nerve fibers**. The median RFS was 8 months in patients with NF compared to 10 months in patients without NF (p=0.800 log rank). **C: Overall survival in patients with late recurrence stratified by nerve fibers**. The median OS was 66 months in patients with NF compared to 78 months in patients without NF (p=0.867 log rank). **D: Recurrence-free survival in patients with late recurrence stratified by nerve fibers.** The median RFS was 34 months in patients with NF compared to 33 months in patients without NF (p=0.697 log rank). RFS, recurrence-free survival; OS, overall survival.

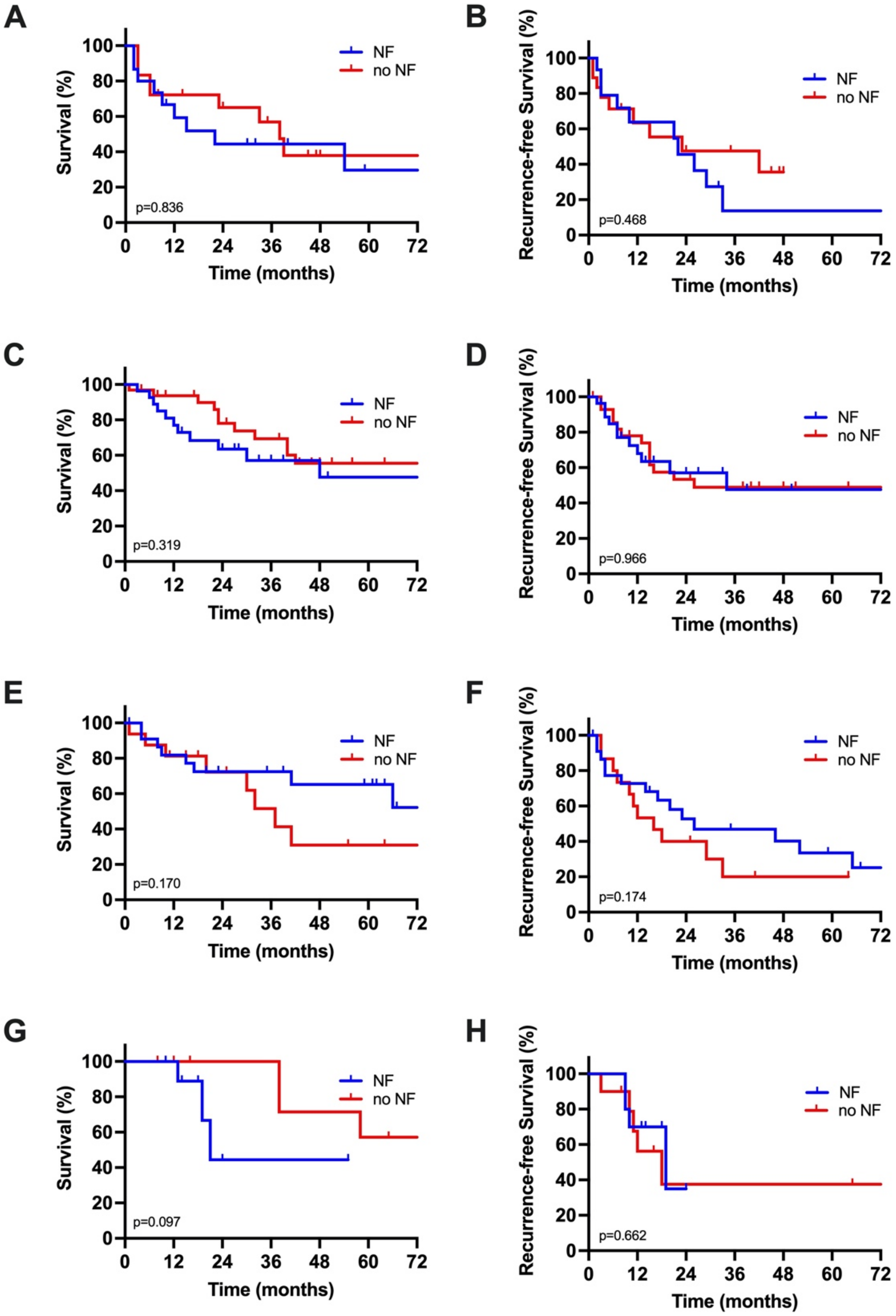

Supplementary Figure S2. Outcome in hepatocellular carcinoma with respect disease etiology. A: Overall survival in patients with alcoholic liver disease stratified by nerve fibers. The median OS was 22 months in patients with NF compared to 38 months in patients without NF (p=0.836 log rank). **B: Recurrence-free survival in patients with alcoholic liver disease stratified by nerve fibers**. The median RFS was 22 months in patients with NF compared to 23 months in patients without NF (p=0.468 log rank). **C: Overall survival in patients with non-alcoholic fatty liver disease stratified by nerve fibers**. The median OS was 48 months in patients with NF compared to n.a. months in patients without NF (p=0.319 log rank). **D: Recurrence-free survival in patients with non-alcoholic fatty liver disease stratified by nerve fibers.** The median RFS was 34 months in patients with NF compared to 26 months in patients without NF (p=0.966 log rank). **E: Overall survival**

in patients with viral liver disease stratified by nerve fibers. The median OS was 82 months in patients with NF compared to 37 months in patients without NF (p=0.170 log rank). **F: Recurrence-free survival in patients with viral liver disease stratified by nerve fibers.** The median RFS was 26 months in patients with NF compared to 12 months in patients without NF (p=0.174 log rank). **G: Overall survival in patients with cryptogenic/other liver disease stratified by nerve fibers**. The median OS was 21 months in patients with NF compared to n.a. months in patients without NF (p=0.097 log rank). **H: Recurrence-free survival in patients with cryptogenic/other liver disease stratified by nerve fibers.** The median RFS was 19 months in patients with NF compared to 18 months in patients without NF (p=0.662 log rank). ALD, alcoholic liver disease; n.a.; not applicable; NAFLD, non-alcoholic fatty liver disease; RFS, recurrence-free survival; OS, overall survival.
